# Understanding staff perspectives on collaborative quality improvement in the ICU: a qualitative exploration

**DOI:** 10.1186/cc9905

**Published:** 2011-03-11

**Authors:** KN Dainty, DC Scales, B Hales, T Sinuff, M Zwarenstein

**Affiliations:** 1 St Michael's Hospital, Toronto, Canada; 2 Sunnybrook Health Sciences Centre, Toronto, Canada

## Introduction

Despite extensive use of QI collaboratives in healthcare and in critical care, little is known about the impact of the collaborative approach on how the intervention is taken up within participating organizations. This in-depth qualitative study investigates the frontline critical care staff perspective on being involved in a large collaborative network for ICU quality improvement.

## Methods

One-on-one key informant interviews were conducted with 32 staff members from a sample of the ICUs who participated in the Ontario ICU Best Practice Collaborative between 2006 and 2008. Using a grounded theory approach, open coding was completed by two qualitative researchers. The open codes were then grouped in to broad theme-oriented categories and all text segments belonging to the same category were then compared. The theme-oriented categories became further refined and formulated into fewer analytic categories through an inductive, iterative process of going back and forth between the data and the analytic framework of the study.

## Results

This research reveals that frontline staff do not feel the need for their unit to be 'like' high-performing peer organizations; they feel that belonging to a collaborative provides the chance to be recognized for providing a high level of care despite their inequalities. The existing QI communication structure within ICUs is highly ineffective for staff engagement, and a QI bubble seems to exist in terms of knowledge transfer. Finally, the idea of collaboration is exhibited more internally in increased intra-team cooperation than externally between organizations, where friendly competition is a more prominent driver. A conceptual framework for QI collaborative design is proposed for future testing.

## Conclusions

These findings indicate that QI collaboratives for ICUs may not function by commonly held inter-organizational assumptions of legitimization, communication and collaboration, which may explain typically mediocre results. Hopefully this work can contribute insight into strategies for more effective use of collaborative efforts for healthcare QI and support new perspectives on their design for use in the ICU environment.

